# Risky business? Whether a more accurate picture of risk assessment in food allergic children and young people affects behaviours in school nurses and school first aiders

**DOI:** 10.1186/2045-7022-3-S3-P117

**Published:** 2013-07-25

**Authors:** H Hanna, T Umasunthar, J Warner, R Boyle

**Affiliations:** 1 Department of Paediatrics, Imperial College, London, UK

## Background

The risk of fatal anaphylaxis in a food allergic person is very low, yet the possibility of this catastrophic event is a major focus for both patients and healthcare professionals. It is unclear how healthcare professionals perceive risk of fatal or non-fatal anaphylaxis in the context of food allergy, and whether their perception of risk influences their management of food allergy.

We investigated whether school nurses/first aiders could accurately estimate fatal anaphylaxis risk in children/young people with food allergy, and whether their assessment of risk correlated with their knowledge and management of food allergy in children/young people.

## Methods

Interview-based surveys completed in 30 schools over 6 months included assessment of risk perception in relation to childhood food allergy using a novel risk assessment tool, an assessment of their knowledge of when to administer adrenaline and assessment of their practical ability to use an adrenaline autoinjector.

## Results

Risk perception varied widely but all participants overestimated fatal food anaphylaxis risk more than they overestimated all-cause mortality risk in young people [median log-fold overestimate for all-cause mortality 0.51 (IQR 0.00, 1.48); food anaphylaxis death 1.20 (IQR 0.06, 2.10) p=0.018]. We did not find any association between estimation of anaphylaxis/fatal anaphylaxis risk in a food allergic child, and practical ability to use an adrenaline autoinjector in a simulated scenario. We did however find that participants who state that they would (unnecessarily) dial emergency services for a non-anaphylactic reaction, also significantly overestimated risk of anaphylaxis in a food allergic person (median log-fold overestimate 1.76 (IQR 0.92, 2.33) vs. 0.72 (IQR -1.51, 1.59) for those who would not dial emergency services for a non-anaphylactic reaction (p=0.009).

Multivariate analyses showed weak evidence that personal or previous medical experience of allergic disease correlates with increased overestimation of fatal food anaphylaxis risk.

## Conclusion

School nurses/first aiders overestimate fatal food anaphylaxis risk more than overestimating other fatality risks in children. The data suggests that this overestimation of risk is not associated with improved ability to recognise/manage anaphylaxis. These data suggest that correcting any risk misperception among healthcare providers, in relation to risk of anaphylaxis in food allergy, is unlikely to be harmful.

## Disclosure of interest

None declared.

